# The effect of serum biochemical parameters on clinical prognosis in children presenting with diabetic ketoacidosis

**DOI:** 10.1590/1806-9282.20240242

**Published:** 2024-07-19

**Authors:** Gunes Isik, Can Aydin

**Affiliations:** 1 Adiyaman University, Faculty of Medicine, Pediatric Nephrology – Adıyaman, Turkey.; 2 Balıkesir City Hospital, Pediatric Endocrinology – Balıkesir, Turkey.

**Keywords:** Child, Biochemical parameters, Diabetic ketoacidosis, Resolution time

## Abstract

**OBJECTIVE:**

The aim of this study was to determine whether diabetes mellitus has a high risk of diabetic ketoacidosis-related complications. Biochemical parameters affect the resolution time of diabetic ketoacidosis.

**METHODS:**

The present study is based on a retrospective evaluation of the records of patients who presented to the Pediatrics Clinic of Adiyaman University Hospital between January 1, 2017, and October 1, 2022, with a diagnosis ofdiabetic ketoacidosis. The demographic characteristics, serum biochemical parameters, blood gas results, and time to transition to subcutaneous insulin therapy were all recorded.

**RESULTS:**

This study included 49 (49%) female and 51 (51%) male patients aged 1–17 years (mean age: 9.05±4.33 years). The average time to clinical improvement of the sample, that is, transition to subcutaneous insulin therapy, was 21.04±7.8 h. An evaluation of the presence of acute kidney injury based on serum urea and creatinine levels and eGFR values revealed no significant effect on the rate of clinical recovery (respective p-values: p=0.076, p=0.494, and p=0.884). A univariate analysis identified blood glucose (p=0.025), blood gas pH (p<0.001), and blood bicarbonate (p=0.004) values as prognostic factors, while a multivariate analysis revealed pH values had an independent and significant effect on the resolution time of diabetic ketoacidosis.

**CONCLUSION:**

Serum glucose, pH, and bicarbonate levels are the most important determinants of clinical prognosis in patients with diabetic ketoacidosis. These findings can serve as a guide for clinicians in the follow-up and treatment of such patients.

## INTRODUCTION

Type 1 diabetes mellitus (DM) is a chronic metabolic disease that leads to polyuria, polydipsia, and weight loss and is managed with insulin, exercise, and dietary planning^1^. Diabetic ketoacidosis (DKA) is a mortal complication of type 1 DM characterized by acidosis, ketosis, and hyperglycemia and frequently accompanied by dehydration and electrolyte disorders. Previous studies have reported that approximately 30–60% of patients present with DKA at the time of initial diagnosis, while DKA is identified in 7–10% of patients during follow-up^2-4^. Prolonged or complicated DKA can lead to organ dysfunction, prolonged hospitalization, and increased treatment costs^5^. Severe acidemia in DKA leads to decreased cardiac contractility, cardiac arrhythmias, and hemodynamic instability, and is the leading factor affecting clinical prognosis due to the damage sustained by vital organs (brain and kidneys). Acidosis also inhibits the binding of insulin to its receptor and the reduction in ketone formation^5-7^. DKA develops suddenly, progresses rapidly, and is accompanied by life-threatening complications, with mortality rates of 5% reported^8^. The regulation of fluids, electrolytes, and acid-base balance disorders is the primary treatment approach to the management of DKA, among which fluid and electrolyte therapies are organized based on International Society for Pediatric and Adolescent Diabetes (ISPAD) guidelines^9^. The management, follow-up, and treatment of DKA are important for the long-term prognosis of patients, given the potential adverse effect on vital organs. The present study investigates the effect of biochemical parameters on DKA resolution time, that is, clinical improvement, in pediatric patients and evaluates the approaches used in its follow-up and treatment. The study further seeks to identify the patient population associated with worse prognosis. Determining the parameters affecting clinical prognosis will contribute to the search for new target drugs for the treatment of DKA.

## METHODS

This study included patients under the age of 18 years who applied to the Pediatrics Clinic of Adiyaman University Hospital between January 1, 2017, and October 1, 2022, with a new diagnosis of type 1 DKA, whose records were evaluated retrospectively. DKA diagnoses were based on ISPAD guidelines, specifically on the presence of hyperglycemia (blood glucose>200 mg/dL), metabolic acidosis (venous blood gas pH<7.3 and/or plasma bicarbonate<18 mmol/L), and the presence of ketonemia and ketonuria^1^. Demographic characteristics, complete blood count, urine density, serum biochemical parameters, blood gas results, time to transition to subcutaneous insulin therapy, and results of renal function tests were all recorded. Patients with chronic metabolic, nephrological, or neurological diseases and those on drugs that may cause fluid-electrolyte or acid-base balance disorders were excluded from the study. The ISPAD guidelines were used as the basis for fluid therapy, and volume expansion was performed with one or more boluses of 0.9% saline infused over 20–30 min until peripheral perfusion was restored. Maintenance and deficit therapy were administered with 0.45% saline over 24–48 h.

### Calculations

Anion gap, corrected sodium, and blood osmolarity were estimated using the following formulas (1):


$$\text{Anion gap}=\text{Na}-\left(\text{Cl}+{\text{HCO}}_{3}\right)\text{: mean value:}12\pm 2\text{mmol/L}.$$

Corrected sodium=measured Na+1.6 ([plasma glucose–5.6]/5.6) mmol/L or measured Na+1.6 ([plasma glucose–100]/100) mg/dL.

Effective osmolality (mOsm/kg)=2×(plasma Na)+plasma glucose mmol/L; normal range is 275–295 mOsm/kg.

### Diabetic ketoacidosis resolution time/transition to subcutaneous insulin therapy

DKA resolution is defined as pH ≥ 7.30, serum bicarbonate >18 mmol/L, and resolution of ketosis or ketonemia^1^. Time to transition to subcutaneous insulin therapy within 24 h of hospitalization was indicated.

### Statistical analysis

IBM SPSS Statistics for Windows (Version 24.0; Armonk, NY: IBM Corp.) was used for the statistical evaluation of the data obtained in the study. Frequency values were used to define the gender distribution of the patients examined in the study, as were mean and standard deviation values for all other parameters. Pearson's correlation coefficient was used for the assessment of the relationship between the parameters and the recovery time of the patients. The optimum cutoff values for glucose, blood gas pH, blood gas bicarbonate, blood gas CO_2_, and serum osmolarity were determined based on the receiver operating characteristic (ROC) curve and the area under the curve (AUC), and median values were used for all other laboratory parameters, with cutoff values used for the categorization of the two groups as "low" or "high." Univariate and multivariate logistic regression analyses were used to determine predictors of patients who recovered within the first 24 h of starting treatment. Variables with significant differences between the treatment-responsive and non-responder groups were included in the logistic regression analysis. All continuous variables were categorized according to clinically identified thresholds. The odds ratio (OR) was reported with corresponding 95% confidence intervals (95%CI). The ROC curve and the area under the ROC curve (ROC-AUC) were calculated for the comparison of independent prognostic factors. The results were accepted as statistically significant within confidence limits of 99% (p<0.01) and 95% (p<0.05).

### Ethics

The study was granted appropriate Institute Review Board approval and was conducted with the approval of the Ethics Commission of the Adiyaman University Hospital in Adiyaman (No. 2022/8-2), in accordance with the Declaration of Helsinki.

## RESULTS

This study included 49 (49%) female and 51 (51%) male patients aged 1–17 years (mean age: 9.05±4.33 years). The mean body mass index of patients was 16.93±3.03 kg/m^2^. Consistent with a diagnosis of DKA, elevated blood glucose, metabolic acidosis, elevated white blood cell counts, prerenal renal failure, and increased urine densities were observed in the sample. The mean time to clinical improvement, that is, the transition to subcutaneous insulin therapy, was 21.04±7.8 h. The laboratory values of the patients are presented in Table 1. None of the patients with prerenal acute kidney injury required renal replacement therapy (hemodialysis/peritoneal dialysis).

**Table 1:** Laboratory test results of the patients.

Laboratory parameters	Min	Max	Mean	SD
Glucose (mg/dL)	238	800	529.75	149.53
BUN (mg/dL)	12	61	27	9.51
Creatinine (mg/dL)	0.20	1.72	0.88	0.26
eGFR (mL/min/1.73 m^2^)	32.65	337.48	73.61	33.44
Na (mmol/L)	120	143	132.37	4.56
Corrected Na (mmol/L)	128.78	150.20	139.24	4.09
K (mmol/L)	3.10	6.10	4.34	0.61
Cl (mmol/L)	88	120	103.05	5.95
Mg (mg/dL)	1.65	2.10	1.84	0.14
pH (mmol/L)	6.78	7.31	7.13	0.12
HCO_3_ (mEq/L)	2.40	18	9.57	3.61
CO_2_ (mEq/L)	2.70	45	22.62	7.11
Anion gap (mmol/L)	6	39.50	24.08	5.75
Urine density	1010	1059	1032.69	8.98
WBC (×10^9^/L)	36	39190	12437.26	6320.84
Serum osmolarity (mOsm/kg)	275.83	333.66	03.81	10.98
HbA1c (%)	7.40	18.10	12.67	2.34
Insulin (μU/mL)	0.18	60.70	4.85	9.25
C-Peptide (ng/mL)	0.10	1.65	0.31	0.28
Time to resolution (h)	6	50	21.04	7.86

SD: standard deviation; WBC: white blood cells.

### Correlation between diabetic ketoacidosis resolution rates and laboratory parameters

A correlation analysis was performed to examine the relationship between DKA resolution rates and laboratory parameters, and the resulting resolution rates were found to be weakly correlated with blood glucose (r:0.201, p:0.045<0.05) levels and anion gap values (r:0.264, p:0.008<0.01); moderately correlated with blood gas pH (r:-0.588, p:0.001<0.01) and HCO_3_ (r:-0.552, p:0.001<0.01) levels; weakly and directly correlated with white blood cell counts (r:0.262, p:0.008<0.01) and serum osmolarity (r:0.219, p:0.029<0.05); and weakly but inversely correlated with CO_2_ (r:-0.371, p:0.001<0.01). As the glucose, anion gap, white blood cell count, and serum osmolarity values increased, so did the time to clinical recovery, whereas as pH and HCO_3_ values increased, the time to clinical recovery decreased.

### Regression analysis results

An analysis of the time to transition to subcutaneous insulin therapy revealed DKA resolution rates both within and after 24 h of hospitalization, with 32 (32%) patients recorded as transitioning to subcutaneous insulin therapy within the first 24 h.

### Parameters cutoff values

**Figure 1:**
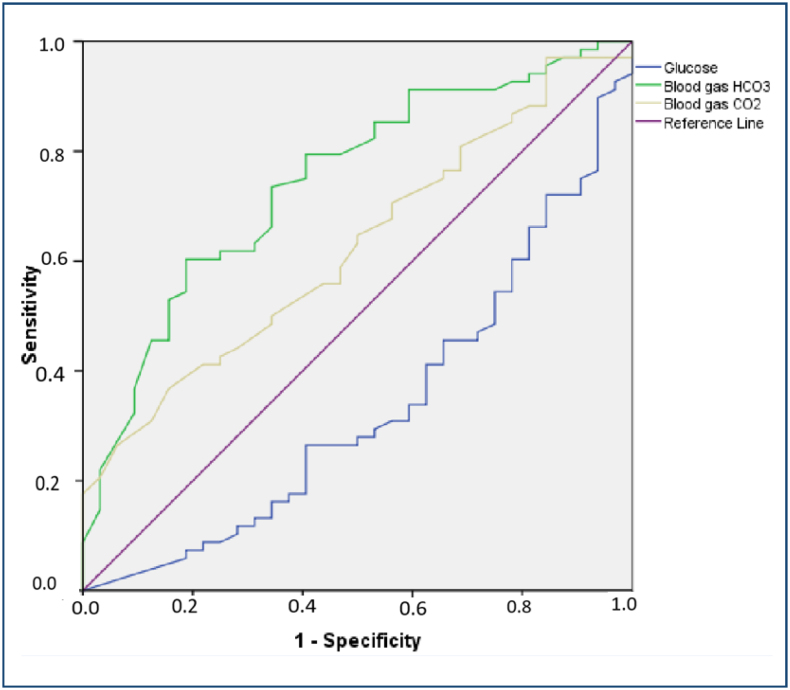
Receiver operating characteristic curve analysis for the predictors of the treatment response within the first 24 h after hospitalization.

In the ROC-AUC analysis, the optimal cutoff and AUC values within the respective confidence intervals for glucose, blood gas pH, blood gas bicarbonate, blood gas pCO_2_, anion gap, and serum osmolarity shown in Table 2 were recorded. Median values were used for all other parameters for which cutoff values could not be determined from the ROC curve analysis (Figure 1).

**Table 2:** Univariate and multivariate analysis for factors affecting response to treatment within the first 24 h of hospitalization.

Variables		Category	Univariate analysis		Multivariate analysis	
			OR (95% CI)	p	OR (95% CI)	p
Age		<9.1/≥9.1	0.61 (0.26–1.43)	0.252		
Sex		Female/male	0.54 (0.23–1.27)	0.157		
Weight (kg)		<28/≥28	0.65 (0.28–1.51)	0.313		
Height (cm)		<133.3/≥133.3	0.65 (0.28–1.51)	0.321		
Laboratory parameters						
	Glucose (mg/dL)	<527.5/≥527.5	2.69 (1.13–6.41)	**0.025**	2.48 (0.97–6.31)	0.057
	BUN (mg/dL)	<26/≥26	2.08 (0.89–4.87)	0.093		
	Creatinine (mg/dL)	<0.84/≥0.84	0.88 (0.42–1.81)	0.718		
	eGFR (mL/min/1.73 m^2^)	<66.9/≥66.9	0.57 (0.25–1.34)	0.200		
	Corrected Na (mmol/L)	<138.8/≥138.8	1.99 (0.84–7.70)	0.117		
	K (mmol/L)	<4.3/≥4.3	0.80 (0.40–1.60)	0.522		
	Cl (mmol/L)	<104/≥104	1.05 (0.45–2.44)	0.917		
	pH (mmol/L)	<7.13/≥7.13	1.18 (0.07–0.44)	**<0.001**	0.23 (0.07–0.77)	**0.017**
	HCO_3_ (mEq/L)	<8.8/≥8.8	0.27 (0.11–0.65)	**0.004**	0.69 (0.21–2.25)	0.535
	CO_2_ (mEq/L)	<22.9/≥22.9	0.61 (0.26–1.43)	0.259		
	Anion gap (mmol/L)	<24.4/≥24.4	1.74 (0.74–4.09)	0.200		
	Urine density	<1033/≥1033	0.73 (0.31–1.70)	0.460		
	WBC (×10^9^/L)	<11305/≥11305	1.45 (0.62–3.37)	0.392		
	Serum osmolarity (mOsm/kg)	<303.28/≥303.2	2.09 (0.89–4.91)	0.091		
	HbA1c (%)	<12.7/≥12.7	1.44 (0.62–3.34)	0.401		
	Insulin (μU/mL)	<2.4/≥2.4	1.53 (0.64–3.65)	0.342		
	C-Peptide (ng/mL)	<0.22/≥0.22	0.68 (0.29–1.61)	0.380		

Significant p-values are indicated in bold. OR: odds ratio; CI, confidence interval; WBC: white blood cells.

### Regression analysis

A logistic regression analysis was carried out to determine the prognostic factors of patients who recovered within the first 24 h. A univariate analysis revealed glucose (p=0.025), blood gas pH (p<0.001), and blood gas bicarbonate (p=0.004) to be prognostic factors, while in a multivariate model created using the factors found to be prognostic for treatment response time in the univariate analysis, only blood gas pH remained an independent prognostic factor (OR=0.23, 95%CI 0.07–0.77, p=0.017) (Table 2).

## DISCUSSION

Diabetic ketoacidosis is a life-threatening and acute complication of type 1 DM^1^, so determining the parameters affecting clinical prognosis and time to resolution is crucial. In the present study, based on the time to resolution of DKA, that is, the time to subcutaneous insulin therapy, an increase in serum glucose (p=0.045), serum osmolarity (p=0.029), and anion gap (p=0.008) were all found to delay clinical resolution, and a delay in clinical resolution was also identified in cases in which the white blood cell count increased (p=0.008). Ying Wei et al. revealed increased serum osmotic pressure to be an independent risk factor for severe DKA and identified an association between increased blood glucose and osmotic pressure and severe DKA^10^. These findings are similar to those reported in the study by Shaltout et al.^11^ In the present study, elevated blood glucose and osmolarity were found to have a negative effect on clinical prognosis and resolution time.

Guzman et al. identified a significant negative correlation between pH, HCO_3_, and pCO_2_ levels at resolution time and reported the pH value at the time of the initial diagnosis to have a significant and independent effect on the time to resolution in patients^12^. Similarly, in the present study, low pH, bicarbonate, and pCO_2_ values were found to delay clinical recovery based on blood gas parameters (p=0.001). After dividing the patients into two groups based on the occurrence of clinical improvement within the first 24 h and after, glucose (p=0.025), blood gas pH (p<0.001), and blood gas bicarbonate (p=0.004) levels were found to be prognostic factors.

In a multivariate model created using the factors found to be prognostic for treatment response time in the univariate analysis in the present study, only blood gas pH remained as an independent prognostic factor (OR=0.23, 95%CI 0.07–0.77, p=0.017), which can be attributed to the worsening of the clinical prognosis by severe acidemia through damage inflicted on vital organs (brain and kidneys).

Previous studies have reported that acute kidney injury negatively affects prognosis^8,10,13^. Huang et al. reported that metabolic acidosis improved later in patients with severe acute kidney injury (AKI)^14^, which suggests that patients with severe AKI who develop intrinsic AKI due to impaired tubular function and renal inflammation have a poor prognosis^15^. In the present study, when the presence of AKI was evaluated with serum urea and creatinine levels and eGFR values, it was found to have no significant impact on the clinical recovery rate (p=0.076, p=0.494, and p=0.884, respectively). AKI seen in the course of DKA can be attributed to decreased intravascular volume, usually leading to a mild course and rapid resolution. The polyuria and vomiting often associated with hyperglycemia lead to a decrease in intravascular volume. AKI developing after hypovolemia is a prerenal condition that can be attributed to decreased renal perfusion and is reversible with appropriate fluid therapy. In the present study, fluids were given based on the degree of dehydration of the patient (4,000 mL/m^2^/day), and this led to a rapid recovery of dehydration and AKI. This rapid recovery may explain the lack of any significant difference in the clinical improvement of patients with AKI when compared to those without AKI. Similar to the present study, Mishra et al. found no correlation between the severity of DKA and that of AKI^16^, and again, similar to our findings, there have been previous studies reporting that the presence of AKI did not affect the duration of hospitalization or the resolution of metabolic acidosis^16^. In the present study, only the first episode of DKA in patients with newly diagnosed DM was evaluated, and the resolution time was recorded as 21.04±7.8 h, compared to the resolution time of 16.25 h reported by Ying Wei et al^10^. The longer resolution time in the present study than that reported by Ying Wei et al. can be attributed to the fact that only the first episode of DKA was evaluated. Guzman et al. showed that resolution times are prolonged with first-episode DKA^12^.

Studies have shown that an increase in the anion gap worsens the clinical prognosis and is an indicator of extracellular fluid loss and dehydration. Isaac Lazar et al. reported that the DKA resolution time becomes longer as the normalization time of the anion gap is prolonged^17^. In line with the literature, the present study found that clinical recovery took longer time as the anion gap increased (p=0.008).

The early diagnosis and treatment of DKA and the recognition of factors associated with a poor prognosis during follow-up are essential for the reversal of DKA.

## CONCLUSION

To the best of our knowledge, there has been no study in the literature to date comparing the resolution time of DKA within and after the first 24 h of hospital admission and evaluating only the attack at the time of the first diagnosis of DKA in a pediatric age group. The results of the present study reveal that serum glucose, pH value, and serum bicarbonate levels all affect resolution time, and in addition, pH value has an independent and significant effect on clinical prognosis. These prognostic markers are easily accessible and can aid clinicians during patient follow-up and treatment.

## STUDY LIMITATION

The retrospective, single-center design of the present study can be considered a limitation. Multi-center, prospective studies are needed to contribute further to the literature.

## CONSENT TO PARTICIPATE

Written informed consent was garnered from the parents of all those included in the study.

## Data Availability

The authors confirm that all data supporting the findings of this study are included within the article.
